# The distribution and antibiotic-resistant characteristics and risk factors of pathogens associated with clinical biliary tract infection in humans

**DOI:** 10.3389/fmicb.2024.1404366

**Published:** 2024-05-09

**Authors:** Shayan Chen, Wenbin Lai, Xuejing Song, Jiongtang Lu, Jianxin Liang, Hao Ouyang, Weihua Zheng, Jianjun Chen, Zhenggang Yin, Huimin Li, Yong Zhou

**Affiliations:** ^1^Department of Laboratory Science, Binhaiwan Central Hospital of Dongguan, Guangdong, China; ^2^Dongguan Key Laboratory of Accurate Etiological Research on the Pathogenesis of Inflammation and Cancer, Guangdong, China; ^3^Central Laboratory, Binhaiwan Central Hospital of Dongguan, Guangdong, China; ^4^Dongguan Key Laboratory of Precision Medicine, Guangdong, China

**Keywords:** biliary pathogens, Gram-negative bacteria, distribution of bacteria, multidrug-resistant bacteria, risk factors

## Abstract

**Introduction:**

Biliary Infection in patients is a common and important phenomenon resulting in severe complications and high morbidity, while the distributions and drug resistance profiles of biliary bacteria and related risk factors are dynamic. This study explored the characteristics of and risk factors for biliary infection to promote the rational use of antibiotics in clinically.

**Methods:**

Bacterial identification and drug susceptibility testing were completed using the Vitek 2 Compact analysis system. The distribution and antibiotic-resistant characteristics of 3,490 strains of biliary bacteria in patients at Nankai Hospital from 2019 to 2021 were analyzed using Whonet 5.6 and SPSS 26.0 software. We then retrospectively analyzed the clinical data and risk factors associated with 2,340 strains of Gram-negative bacilli, which were divided into multidrug-resistant bacteria (1,508 cases) and non-multidrug-resistant bacteria (832 cases) by a multivariate Cox regression model.

**Results and discussion:**

A total of 3,490 pathogenic bacterial strains were isolated from bile samples, including 2,340 (67.05%) Gram-negative strains, 1,029 (29.48%) Gram-positive strains, and 109 (4.56%) fungal strains. The top five pathogenic bacteria were *Escherichia coli*, *Klebsiella pneumoniae*, *Enterococcus faecium*, *Enterococcus faecalis*, and *Pseudomonas aeruginosa*. The rate of *Escherichia coli* resistance to ciprofloxacin increased (*p* < 0.05), while the resistance to amikacin decreased (*p* < 0.05). The resistance of *Klebsiella pneumoniae* to cephalosporins, carbapenems, *β*-lactamase inhibitors, cephalases, aminoglycosides, and quinolones increased (*p* < 0.05), and the resistance of *Pseudomonas aeruginosa* to piperacillin, piperacillin/tazobactam, ticacillin/clavulanic acid, and amicacin declined significantly (*p* < 0.05). The resistance of *Enterococcus faecium* to tetracycline increased by year (*p* < 0.05), and the resistance of *Enterococcus faecalis* to erythromycin and high-concentration gentamicin declined (*p* < 0.05). Multivariate logistic regression analysis suggested that the administration of third- or fourth-generation cephalosporins was an independent risk factor for biliary infection. In summary, Gram-negative bacilli were the most common pathogenic bacteria isolated from biliary infection patients, especially *Escherichia coli*, and the rates and patterns of drug resistance were high and in constant flux; therefore, rational antimicrobial drug use should be carried out considering risk factors.

## 1 Introduction

Biliary infection in patients is common and has been associated with invasive clinical procedures, septicemia, intestinal barrier dysfunction, and gut bacteria translocation (Chi et al., 2023; Ekpanyapong and Reddy, 2023). It continues to be a significant cause of severe complications and morbidity in patients, particularly in elderly patients with underlying comorbidities. The heightened mortality rate among patients with infected biliary secretions underscores the critical need for timely diagnosis and treatment of biliary tract infections. Therefore, for patients with bile infectious symptoms, bile samples are frequently used for clinical microbiological culture tests. However, the bile bacterial spectrum and antibiotic resistance characteristics are constantly changing. Based on previous literature reports, the widespread use of antibiotics globally has increased the likelihood of bacterial species alterations. Data indicates that pathogenic bacteria such as *Escherichia coli, Enterococcus faecium, Klebsiella pneumoniae, and Serratia*, particularly *Enterococcus faecium*, have become predominant in recent cases of biliary tract infections. It is noteworthy that the trend of drug resistance is worsening (Ruan et al., 2019).

Gram-negative bacilli, which are some of the most common opportunistic pathogens in hospitals, have shown increasing trends toward drug resistance in recent years. Gram-negative bacilli mainly include those in the Enterobacteriaceae family and nonfermentative bacteria, and easily evolve into multidrug-resistant (Chen S. et al., 2021) organisms (MDROs) that are resistant to three or more types of clinical antibiotics used at the same time. In recent years, with the extensive use of antibiotics for the treatment of bacterial infections, MDROs have become common and important pathogens responsible for clinical infections, resulting in an intractable challenge for clinical diagnosis and treatment (Kawanishi et al., 2017); therefore, it is necessary to clarify the risk factors for MDRO infection in patients.

We conducted this study to understand the characteristics of pathogens causing biliary infections and related risk factors in patients, guide clinical detection, promote the rational use of antibiotics, control multidrug-resistant bacterial infections, and improve the cure rate in biliary tract infectious patients. We analyzed the in-hospital data of patients from January 2019 to December 2021 to retrospectively identify the biliary pathogenic spectrum and drug resistance profiles, Gram-negative multidrug-resistant bacteria distributions and resistance rates, and associations of risk factors in the clinical characteristics of the patients. This analysis aimed to provide valuable insights into the changing trends of biliary tract infections and drug resistance, ultimately informing more effective clinical management, improved detection capability, and prevention strategies in the medical future. The study results are as follows.

## 2 Materials and methods

### 2.1 Study subjects

This retrospective cross-sectional study, recruited 1,556 patients, including 962 males and 594 females, who were hospitalized at Tianjin Nankai Hospital, Tianjin Medical University from January 1, 2019, to December 31, 2021. Their data was accessed on January 15, 2022, using the software of the hospital information system and laboratory information system for research purposes within the hospital.

### 2.2 Sample collection

The present study obtained ethical approval (no. NKYY_YXKT_IRB_2021_155_01) from the Ethical Committee of Tianjin Nankai Hospital, Nankai Clinical College, Tianjin Medical University, following the Helsinki Declaration. Due to its retrospective design, a waiver of participant informed consent was granted by the Ethical Committee of Nankai Clinical College, Tianjin Medical University.

The patients were 18–86 years of age (average age = 62.3 ± 23). Specimens were collected from patients presenting symptoms of potential biliary disease or infection who had not been treated with antibiotics. Bacterial culture identification was conducted by directly extracting 2–10 mL of bile from the gall bladder or through bile duct puncture and drainage. Additionally, newer technologies such as ERCP and other ultrasonographic techniques were utilized to aid in collecting bile samples from the suspected site of infection (Zijlstra, 2022).

### 2.3 Sample culture and bacterial detection

Bile samples were aseptically injected into aerobic and anaerobic culture bottles, mixed well, placed in a BacT/Alert3D automated blood culture instrument, and continuously monitored for 2 days (Plantamura et al., 2012). Samples with no positive indication were cultured on a blood agar medium and defined as negative if no bacteria were detected and positive if there was evidence of bacterial proliferation and the instrument was able to detect a yellow color on the bottom indicator of the culture bottles at the indicated time. Positive bile samples were transferred to blood agar, Sabouraud fungal medium, McConkey agar, and chocolate agar (Tianjin Jinzhang Biotechnology Development Co., Ltd. Tianjin, China) and incubated in 371 carbon dioxide bacteria incubators at 35°C and 5% CO_2_ for 18–24 h. Anaerobic culture bags (1.5 L; Qingdao Haibo Biotechnology, Shandong, China) were used for further culture and separation, as needed (Chen S. et al., 2021).

### 2.4 Bacterial strain identification and drug susceptibility testing

Sole pathogenic colonies were obtained and manually sampled using a VITEK 2 compact automatic microbial analysis system and its supporting identification cards to identify strains and determine the drug susceptibility profiles. The Vitek 2 compact automated microbial identifier is capable of automatically identifying over 400 strains of bacteria, including Gram-negative bacilli, Gram-positive bacteria, fungi, aerobic bacilli, and anaerobic bacteria. It can run thirty cartridges simultaneously and perform susceptibility testing using susceptibility cards. Some antimicrobial drug susceptibility testing that failed to complete in the analyzer was conducted using the paper diffusion method, with interpretation following the American CLSI M100 document (2018–2020). The presence of MDRO, ESBL, VRE, and MRSA was determined according to the National Clinical Laboratory Practice (3rd edition). While complete all microbiological identification still relies on conventional biochemical tests and a combination of methods such as Gram stain observation of morphological colonial characters (identifying hemolytic or non-hemolytic colonies on blood agar), lactose or non-lactose colonies on MacConkey’s agar, oxidase test, catalase test, plasma coagulase test and standard biochemical tests application. Carbapenem resistance was defined when the isolate showed non-susceptibility to any tested carbapenems according to Clinical Standards Institute (CLSI) breakpoints (Carvalhaes et al., 2023). All testing was conducted in strict accordance with international clinical operating procedures.

### 2.5 Standard quality control

Standard control strains, *Staphylococcus aureus* ATCC29213, *Escherichia coli* ATCC25922, and *Enterobacter cloacae* ATCC700323 (provided by the clinical testing center at the National Health and Family Planning Commission in China), were tested in parallel with the sample testing (Alreshidi et al., 2023).

### 2.6 Statistical processing methods

The bacterial and drug resistance data were analyzed and statistically processed using the WHONET 5.6 software. Additionally, SPSS 26.0 software (SPSS Inc.) was utilized to conduct the Chi-square test for the drug resistance rates of pathogens among groups isolated from bile specimens during 2019–2021 at a significance level of *α* = 0.05.

### 2.7 Independent risk factors for death were identified using a multivariate cox regression model

In the analysis of risk factors for multidrug resistance, 2,340 cases with Gram-negative bacilli isolation during hospitalization from 2019 to 2021 were divided into a multidrug-resistant bacteria group (MDROs, 1,508 cases) and a non-multidrug-resistant bacteria group (Non-MDROs, 832 cases), and their clinical characteristics were retrospectively analyzed to identify potential risk factors. Independent risk factors for mortality were identified using a multivariate Cox regression model. Group comparisons for categorical variables were assessed using χ 2 tests. The findings were reported with a 95% confidence interval (CIs). Statistical analysis was conducted using SPSS Statistics version 26.0 (SPSS Inc.), with statistical significance set at *p* < 0.05.

## 3 Results

### 3.1 General characteristics of the distributions and proportions of bile pathogenic bacteria

During 2019–2021, the positivity rate of the bile culture samples was 72.30% (1,125/1,556), and mixed infections of two or more bacteria accounted for 92.00% (1,035/1125). A total of 3,490 pathogen strains were isolated, of which 2,340 (67.05%) were Gram-negative, 1,029 (29.48%) were Gram-positive, and 109 (3.12%) were fungal, the typical culture images were included in Supplementary Figure S1.

**Figure 1:**
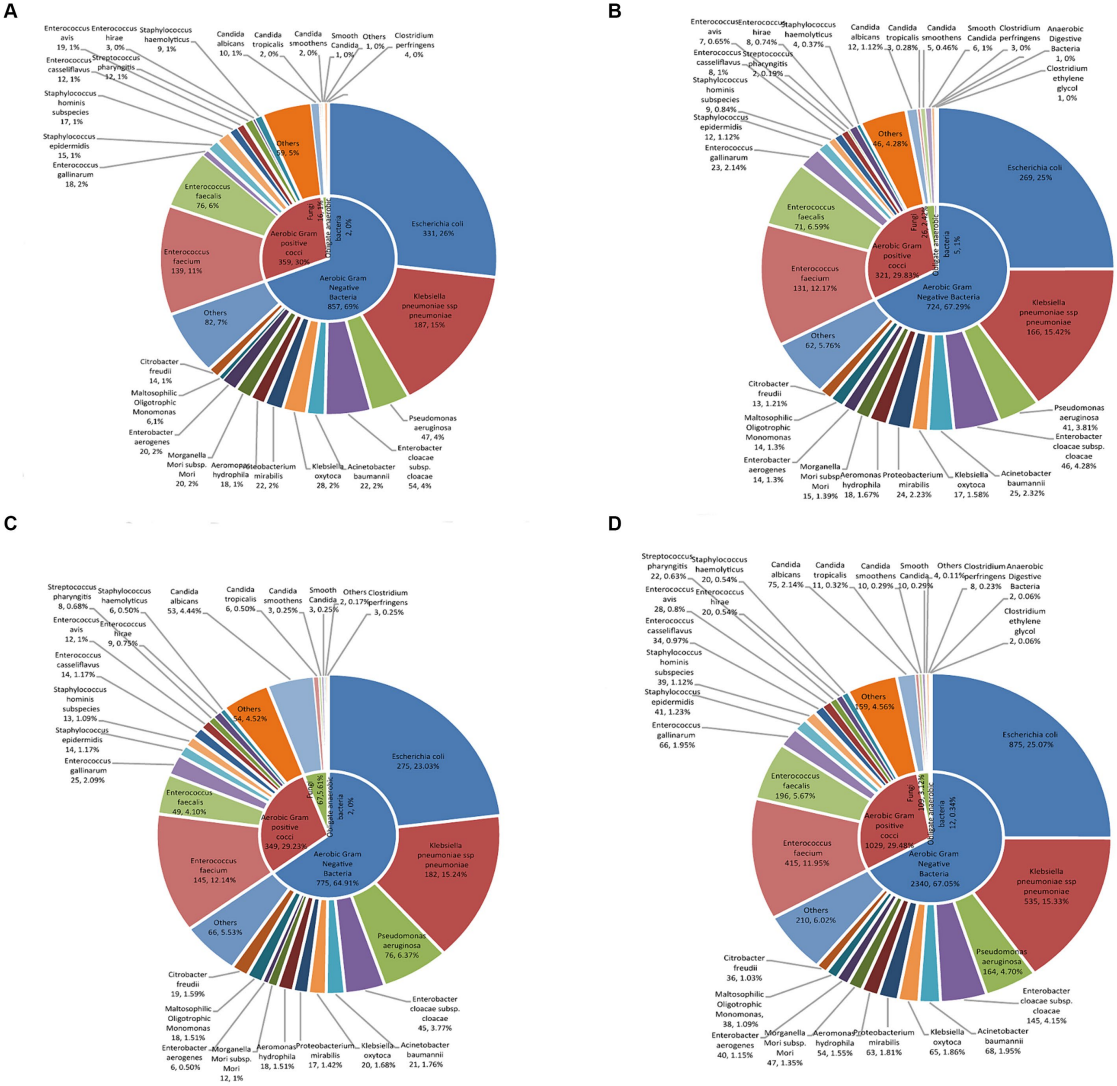
The characteristics of distribution and proportion of pathogenic bacteria in patients with bile tract infection. The distribution and proportion of pathogenic bacteria in patients with bile tract infection in **(A)** 2019, **(B)** 2020, **(C)** 2021, and **(D)** 2019–2021 using double-layered pie charts. The inner layers in the pie charts were classified based on the proportion of aerobic Gram-positive bacteria, aerobic Gram-negative bacteria, fungi, and anaerobic bacteria. The outer pie charts showed the specific bacterial species under their respective categories.

Among the Gram-negative bacteria, *Escherichia coli* had the highest rate of detection (875 strains/25.07%), followed by *Klebsiella pneumoniae* (535 strains/15.33%), *Pseudomonas aeruginosa* (164 strains/4.70%), and *Enterobacter cloacae sewer subspecies* (145 strains/4.15%). Among the Gram-positive bacteria, *Enterococcus faecium* (415 strains/11.95%), *Enterococcus faecalis* (196 strains/5.67%), *Enterococcus gallinae* (66 strains/1.95%), and *Staphylococcus epidermidis* (41 strains/1.23%) had the highest rates of detection. Pathogen composition and distribution by year is shown in Figure 1 and Supplementary Figure S2.

### 3.2 Distribution of pathogenic bacteria in the clinical departments

**Figure 2:**
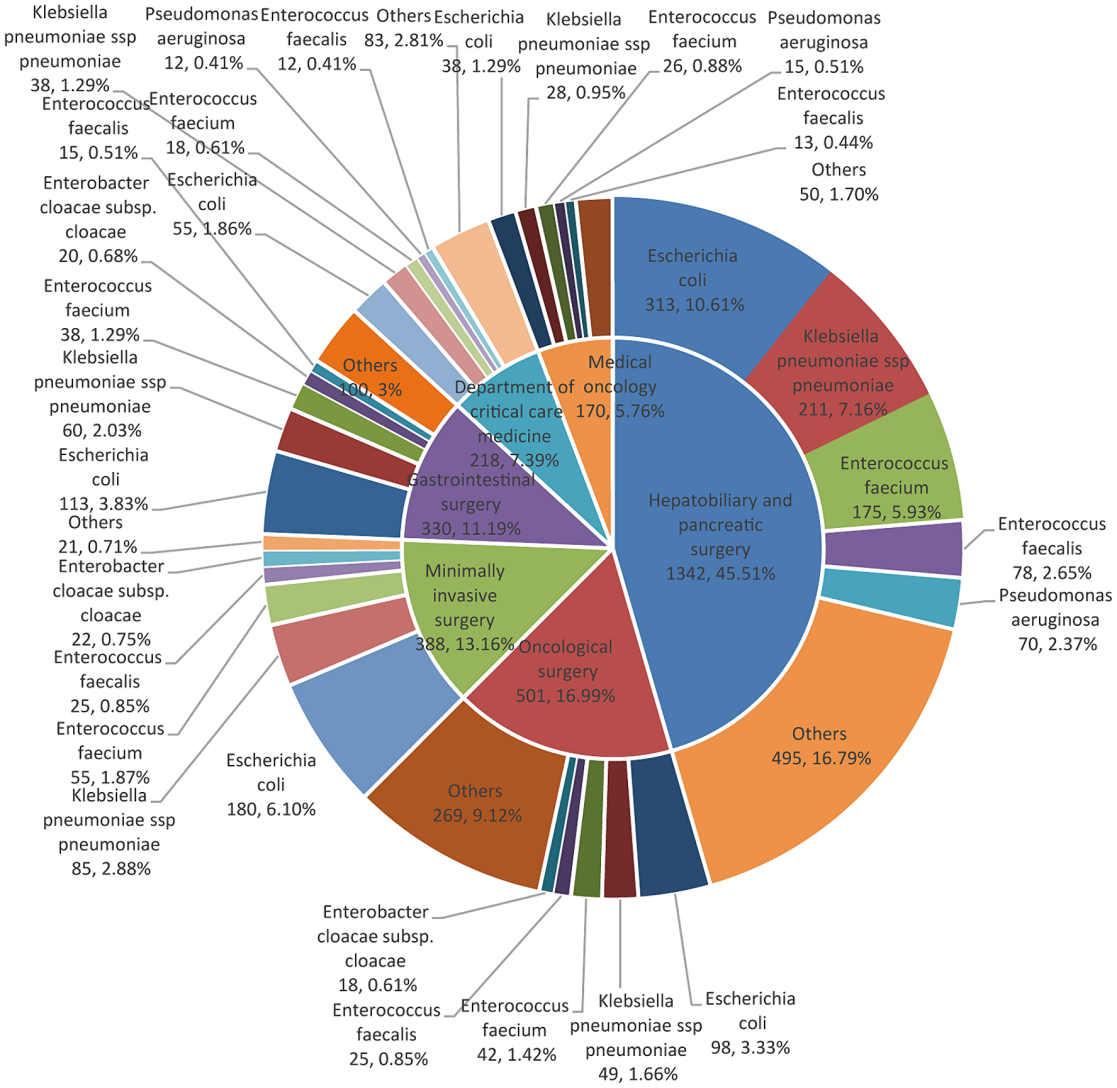
The distribution of major pathogens in patients is seen in each of the top six clinical departments with a pie chart of double layers. The inner layers in the pie charts were classified according to the top six clinical departments. The outer pie charts displayed the top bacterial species under their respective departments.

During 2019–2021, the top six specialties with the highest number of pathogen strains detected in bile samples were (from high to low) the clinically major departments in the hospital: hepatobiliary and pancreatic surgery, surgical oncology, minimally invasive surgery, gastrointestinal surgery, intensive care, and oncology (Figure 2).

### 3.3 Drug-resistant characteristics of the major gram-negative bacteria, *Enterobacteriaceae*

**Figure 3:**
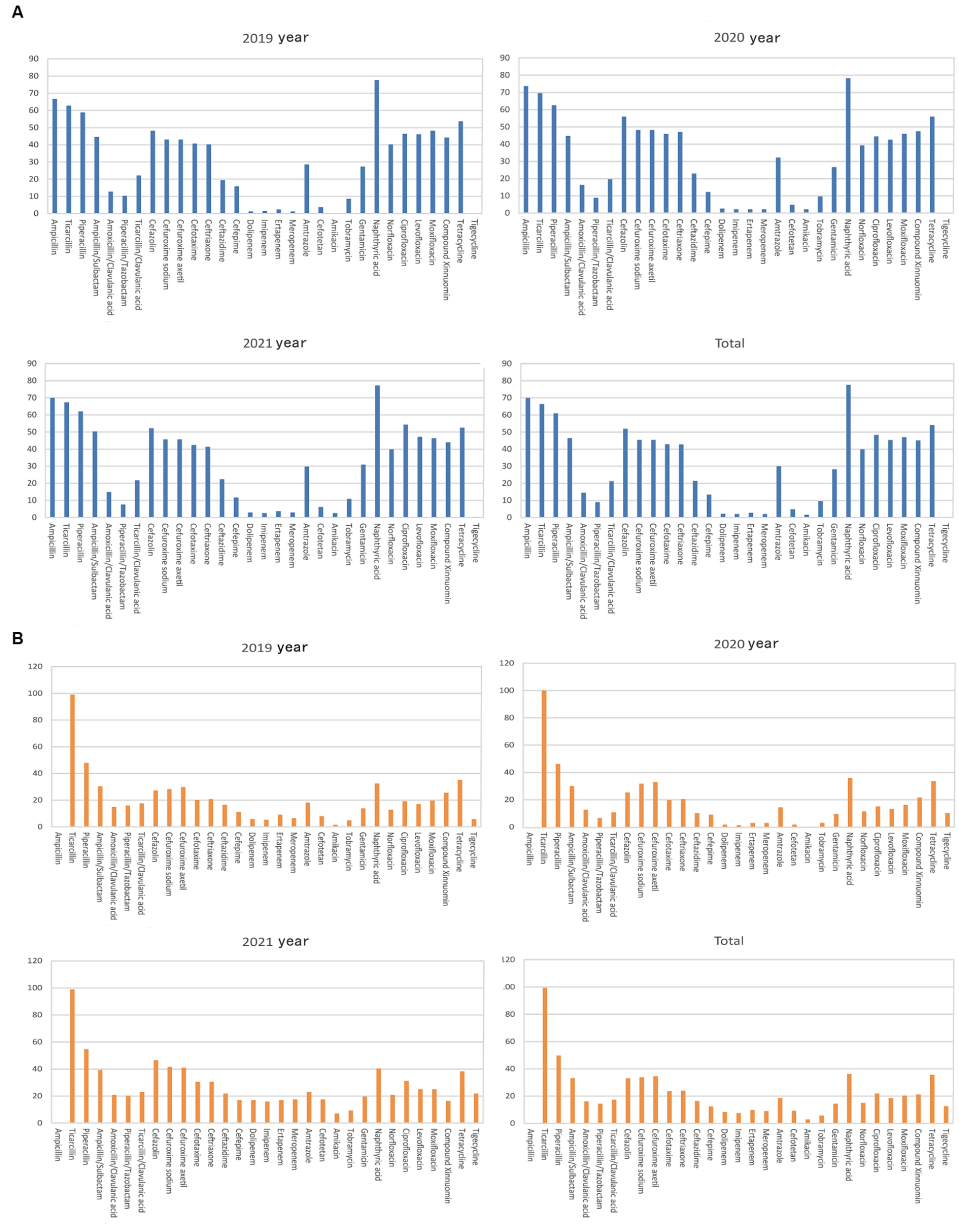
Analysis of the rates of fermenting bacterial drug resistance. On the X-axis of all graphs are antimicrobial agents for Gram-negative bacteria, while the Y-axis displays the percentage of resistant bacterial strains. **(A)** Analysis of *Escherichia coli* drug resistance in bile tract infection patients during 2019–2021. Upper left: *Escherichia coli* drug resistance in 2019. Upper right: *Escherichia coli* drug resistance in 2020. Left bottom: *Escherichia coli* drug resistance in 2021. Right bottom: *Escherichia coli* drug resistance during 2019–2021. **(B)** Analysis of *Klebsiella pneumoniae* drug resistance in patients with bile tract infection patients during 2019–2021. Upper left: *Klebsiella pneumoniae* drug resistance in 2019. Upper right: *Klebsiella pneumoniae* drug resistance in 2020. Left bottom: *Klebsiella pneumoniae* drug resistance in 2021. Right bottom: *Klebsiella pneumoniae* drug resistance during 2019–2021.

The detection rate of extended-spectrum beta-lactamases (ESBLs) in *Escherichia coli* was 37.21%. The rate of *Escherichia coli* resistance to antibiotics remained stable during 2019–2021 (Figure 3A and Table 1), and the average rate of resistance to antibiotics, including ampicillin, ticacillin, piperacillin, ampicillin/sulbactam, cefazolin, cefuroxime sodium, cefuroxime axetil, cefotaxime, ceftriaxone, nalidixic acid, levofloxacin, ciprofloxacin, moxifloxacin, tetracycline, and cotrimoxazole, was >40%. Interestingly, the rate of resistance to ciprofloxacin (42.03–56.20%) increased significantly in 2021 (*p* < 0.05) (Figure 3A and Table 1). Meanwhile, the rates of *Escherichia coli* resistance to amoxicillin/clavulanic acid, ticacillin/clavulanic acid, ceftazidime, cefepime, amtronam, and gentamicin, were relatively low. *Escherichia coli* was more sensitive to piperacillin/tazobactam, dolipenem, imipenem, ertapenem, meropenem, cefotetan, tobramycin, amikacin and tigecycline, with average drug resistant rates of 10.29, 3.29, 1.94, 3.05, 1.92, 5.09, 9.25, 2.05 and 0.23%, respectively.

**Table 1:** The analysis revealed the resistance rate of *Escherichia coli* to antibiotics.

Antibiotics	*χ* ^2^	*p*
Ampicillin	3.5	0.17
Ticarcillin	3.4	0.19
Piperacillin	1.1	0.59
Ampicillin/Sulbactam	2.5	0.29
Amoxicillin/Clavulanic acid	1.6	0.46
Piperacillin/Tazobactam	1.3	0.51
Ticarcillin/Clavulanic acid	0.6	0.74
Cefazolin	3.6	0.17
Cefuroxime sodium	1.6	0.46
Cefuroxime axetil	1.6	0.46
Cefotaxime	1.7	0.42
Ceftriaxone	3.1	0.22
Ceftazidime	1.4	0.51
Cefepime	2.7	0.26
Dolipenem	2.3	0.31
Imipenem	0.8	0.66
Ertapenem	1.2	0.55
Meropenem	2.2	0.34
Amtrazole	1	0.61
Cefotetan	2.1	0.35
Amikacin	5.7	0.06
Tobramycin	1	0.61
Gentamicin	1.4	0.5
Naphthyric acid	0.1	0.96
Norfloxacin	0.1	0.97
Ciprofloxacin	6.1	<0.05^*^
Levofloxacin	1.2	0.54
Moxifloxacin	0.4	0.84
Compound Xinnuomin	0.9	0.65
Tetracycline	0.7	0.72
Tigecycline	2.2	0.34

SPSS 26.0 software was employed to conduct the Chi-square test for the drug resistance rates of *Escherichia coli* isolated from bile specimens during 2019–2021 at a significance level of *α* = 0.05. The symbol * indicates statistical significance.

Except for tigecycline and carbapenem antibiotics, doripenem, imipenem, ertapenem, and meropenem, *Klebsiella pneumoniae* had a higher sensitivity than *Escherichia coli* to most antibiotics (Figure 3B and Table 2). The average rates of *Klebsiella pneumoniae* resistance to these drugs were 11.34, 8.25, 7.76, 10.02, and 8.94%, respectively, increasing significantly in 2021 (*p* < 0.01). The rates of *Klebsiella pneumoniae* resistance to piperacillin/tazobactam, ticacillin/clavulanate, cefazolin, cefuroxime sodium, cefotaxime, ceftriaxone, ceftazidime, cefotetan, amicacin, tobramycin, gentamicin, norfloxacin, and ciprofloxacin showed an increasing trend (*p* < 0.05). Finally, the rate of ESBL detection in *Klebsiella pneumoniae* was 17.56%.

**Table 2:** The analysis of the resistance rate of *Klebsiella pneumoniae* to antibiotics.

Antibiotics	*χ* ^2^	*p*
Ampicillin	-	-
Ticarcillin	1.8	0.4
Piperacillin	2.9	0.23
Ampicillin/Sulbactam	4.6	0.1
Amoxicillin/Clavulanic acid	4.7	0.1
Piperacillin/Tazobactam	13.6	<0.01*
Ticarcillin/Clavulanic acid	9.1	0.01*
Cefazolin	22.5	<0.01*
Cefuroxime sodium	7.8	0.02*
Cefuroxime axetil	5.4	0.07
Cefotaxime	7.5	0.02*
Ceftriaxone	6.7	0.04*
Ceftazidime	8.7	0.01*
Cefepime	5.5	0.06
Dolipenem	28.5	<0.01*
Imipenem	28.8	<0.01*
Ertapenem	19.3	<0.01*
Meropenem	24.7	<0.01*
Amtrazole	4.3	0.12
Cefotetan	26.1	<0.01*
Amikacin	17.2	<0.01*
Tobramycin	6.9	0.03*
Gentamicin	7.3	0.03*
Naphthyric acid	2.6	0.28
Norfloxacin	7.2	0.03*
Ciprofloxacin	14.7	<0.01*
Levofloxacin	8.7	0.01*
Moxifloxacin	4.4	0.11
Compound Xinnuomin	4.7	0.1
Tetracycline	0.9	0.64
Tigecycline	22.8	<0.01*

SPSS 26.0 software was utilized to conduct the Chi-square test for the drug resistance rates of *Klebsiella pneumoniae* isolated from bile specimens during 2019–2021 at a significance level of *α* = 0.05. “-” indicates that some test results were incomplete due to unavoidable problems such as technical updates or detection problems. *means statistically significant.

### 3.4 Drug resistance of the non-fermenting bacteria, *Pseudomonas aeruginosa*

**Figure 4:**
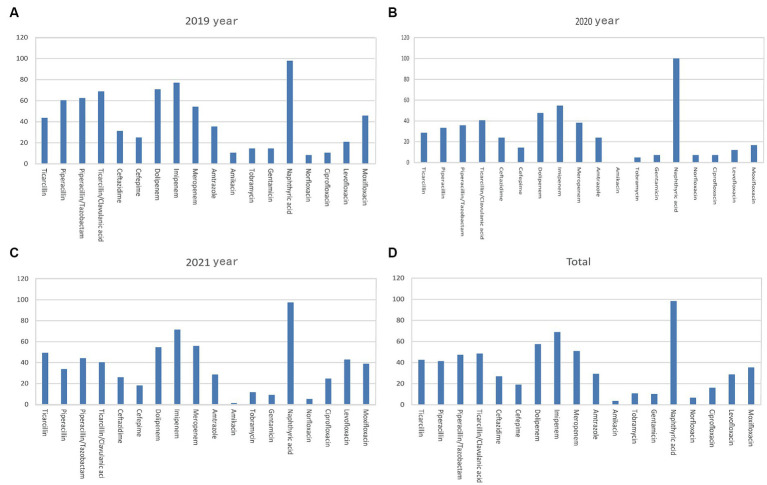
Analysis of the rates of non-fermenting bacterial drug resistance. Rates of *Pseudomonas aeruginosa* drug resistance in **(A)** 2019, **(B)** 2020, **(C)** 2021, and **(D)** 2019–2021. The X-axis of all the graphs displays antimicrobial agents, while the Y-axis represents the percentage proportions of resistant bacteria strains.

*Pseudomonas aeruginosa* was the primary non-fermenting bacteria detected in the bile samples, with a higher average 3-year rate of resistance to carbopenicillin antibiotics (>50%) than all the Gram-negative pathogens (Figure 4 and Table 3). Meanwhile, the rates of *Pseudomonas aeruginosa* resistance to piperacillin, piperacillin/tazobactam, ticacillin/clavulanate, and amikacin decreased significantly over time (*p* < 0.05). The rates of resistance to quinolone antibiotics, such as ciprofloxacin, levofloxacin, and moxifloxacin, declined in 2020 and rebounded to varying degrees in 2021 (*p* < 0.05).

**Table 3:** The analysis of the resistance rate of *Pseudomonas aeruginosa* to antibiotics.

Antibiotics	*χ* ^2^	*p*
Ticarcillin	4.8	0.09
Piperacillin	10.1	<0.01^*^
Piperacillin/Tazobactam	7	0.03^*^
Ticarcillin/Clavulanic acid	11.1	<0.01^*^
Ceftazidime	0.7	0.71
Cefepime	1.7	0.42
Dolipenem	5.4	0.07
Imipenem	5.6	0.06
Meropenem	3.7	0.16
Amtrazole	1.5	0.47
Amikacin	9.2	0.01^*^
Tobramycin	2.4	0.31
Gentamicin	1.5	0.46
Naphthyric acid	1.1	0.59
Norfloxacin	0.5	0.78
Ciprofloxacin	7.8	0.02^*^
Levofloxacin	14.8	<0.01^*^
Moxifloxacin	9.2	0.01^*^

SPSS 26.0 software was employed to conduct the Chi-square test for the drug resistance rates of *Pseudomonas aeruginosa* isolated from bile specimens during 2019–2021 at a significance level of *α* = 0.05. *means statistically significant.

### 3.5 Drug-resistant characteristics of the major gram-positive bacteria

**Figure 5:**
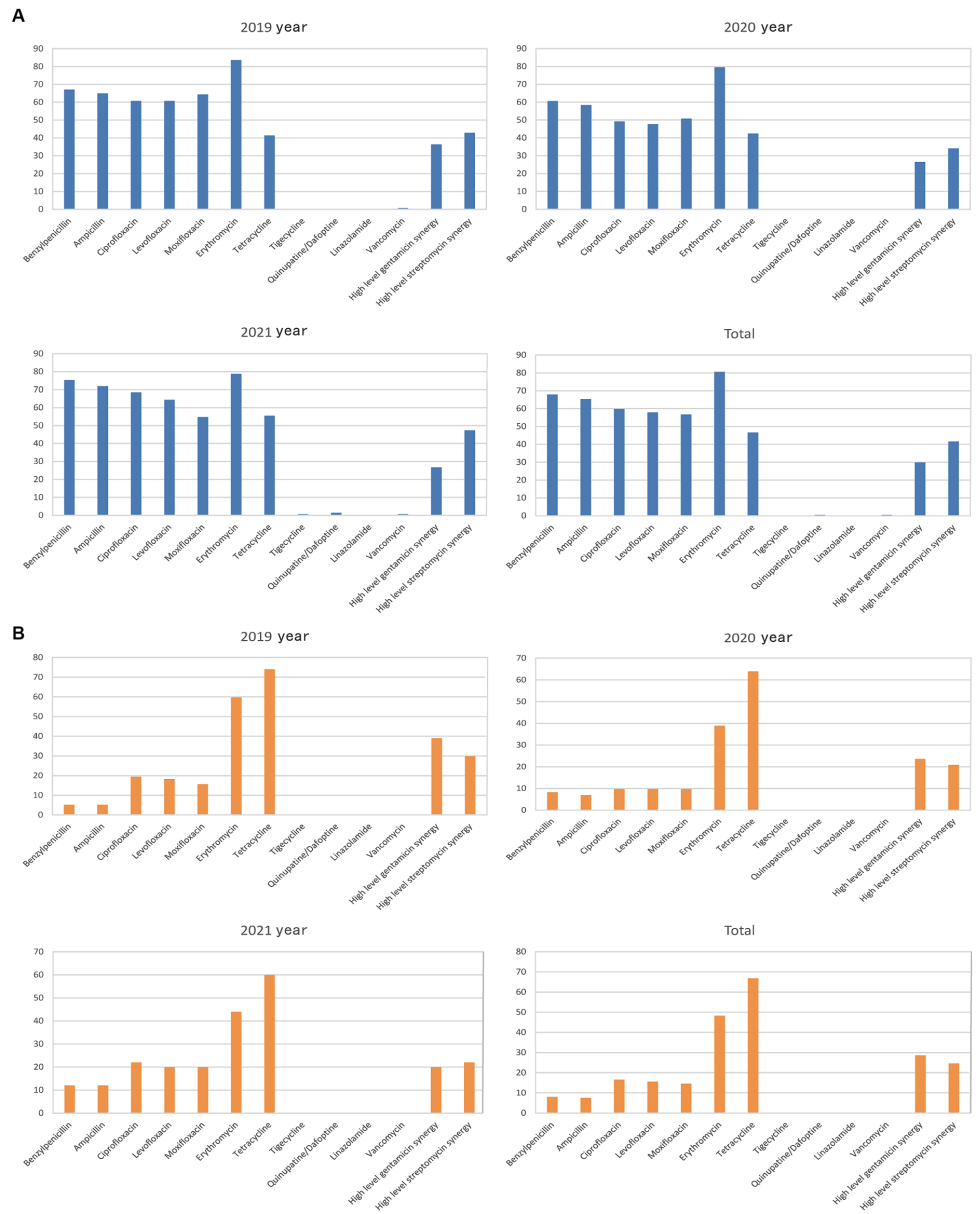
Characteristics of major Gram-positive bacterial drug resistance. The X-axis of all graphs represents antimicrobial agents for Gram-positive bacteria, while the Y-axis depicts the percentages of resistant bacterial strains. **(A)** Analysis of *Enterococcus faecium* drug resistance in patients with bile tract infection during 2019–2021. Upper left: *Enterococcus faecium* drug resistance in 2019. Upper right: *Enterococcus faecium* drug resistance in 2020. Left bottom: *Enterococcus faecium* drug resistance in 2021. Right bottom: *Enterococcus faecium* drug resistance during 2019–2021. **(B)** *Enterococcus faecalis* drug resistance in patients with bile tract infection during 2019–2021. Upper left: *Enterococcus faecalis* drug resistance in 2019. Upper right: *Enterococcus faecalis* drug resistance in 2020. Left bottom: *Enterococcus faecalis* drug resistance in 2021. Right bottom: *Enterococcus faecalis* drug resistance during 2019–2021.

The rate of *Enterococcus faecium* resistance to a variety of antimicrobials, including benzicillin, ampicillin, levofloxacin, ciprofloxacin, moxifloxacin, and erythromycin, surpassed 50%, and resistance rates to benzicillin, levofloxacin, and ciprofloxacin varied over time (*p* < 0.05) (Figure 5A and Table 4). There was an increase in the rate of *Enterococcus faecium* resistance to tetracycline by year (*p* < 0.05). *Enterococcus faecium* also showed high sensitivity to tigecycline and vancomycin, with average resistance rates of 0.22 and 0.52%, respectively. Fortunately, no linezolidin-resistant *Enterococcus faecium* strains were identified.

**Table 4:** The analysis of the resistance rate of *Enterococcus faecium* to antibiotics.

Antibiotics	*χ* ^2^	*p*
Benzylpenicillin	7	0.03*
Ampicillin	5.7	0.06
Ciprofloxacin	10.8	<0.01*
Levofloxacin	8.6	0.01*
Moxifloxacin	5.4	0.07
Erythromycin	1.2	0.55
Tetracycline	7.1	0.03*
Tigecycline	1.9	0.39
Quinupatine/Dafoptine	3.7	0.15
Linazolamide	-	-
Vancomycin	0.9	0.63
High level gentamicin synergy	4.3	0.12
High level streptomycin synergy	5.1	0.08

SPSS 26.0 software was utilized to conduct the Chi-square test for the drug resistance rates of *Enterococcus faecium* isolated from bile specimens during 2019–2021 at a significance level of *α* = 0.05. * denotes statistical significance. “-” indicates that some test results were incomplete due to unavoidable problems such as technical updates or detection problems.

*Enterococcus faecalis* was more sensitive to most antibiotics than *Enterococcus faecium*, and showed variability in its resistance to erythromycin (*p* < 0.05). In contrast, the rate of resistance to high-concentration gentamicin declined by year (*p* < 0.05) (Figure 5B and Table 5).

**Table 5:** The analysis of the resistance rate of *Enterococcus faecalis* to antibiotics.

Antibiotics	*χ* ^2^	*p*
Benzylpenicillin	1.9	0.38
Ampicillin	2.1	0.36
Ciprofloxacin	4	0.14
Levofloxacin	3	0.22
Moxifloxacin	2.6	0.27
Erythromycin	7	0.03*
Tetracycline	3.1	0.21
Tigecycline	-	-
Quinupatine/Dafoptine	-	-
Linazolamide	-	-
Vancomycin	-	-
High level gentamicin synergy	6.7	0.04*
High level streptomycin synergy	1.9	0.4

SPSS 26.0 software was employed to conduct the Chi-square test for the drug resistance rates of *Enterococcus faecalis* isolated from bile specimens during 2019–2021 at a significance level of *α* = 0.05. *means statistically significant. “-” indicates that some test results were incomplete due to unavoidable problems such as technical updates or detection problems.

### 3.6 Drug resistance of major fungal pathogens

*Candida albicans* and *Candida tropicalis* were the major fungi isolated from the bile samples, and *Candida albicans* showed 100.00% sensitivity to common antifungal drugs during 2019–2021. Meanwhile, *Candida tropicalis* showed partial or full resistance to itraconazole, fluconazole, and voriconazole in 2019 and 2021, respectively. Importantly, *Candida tropicalis* showed no resistance to other antifungal drugs (Table 6).

**Table 6:** The analysis of the resistance rate of major fungi detected in bile tract infection from 2019 to 2021 year (%).

Antibiotics	*Candida albicans*				*Candida tropicalis*			
	2019 year	2020 year	2021 year	Total	2019 year	2020 year	2021 year	Total
	(*n* = 10)	(*n* = 12)	(*n* = 53)	(*n* = 75)	(*n* = 2)	(*n* = 3)	(*n* = 6)	(*n* = 11)
Amphotericin B	0	0	0	0	0	0	0	0
Fluconazole	0	0	0	0	0	0	16.67	10
Voriconazole	0	0	0	0	0	0	16.67	10
5-fluorocytosine	0	0	0	0	0	0	0	0
Itraconazole	0	0	0	0	50	0	16.67	20

The fungus drug resistance data was analyzed and statistically processed using WHONET 5.6 software. The data in the table represented the percentages of resistance rates of major fungi for the various antibiotics in different years.

### 3.7 Analysis of major risk factors

Analysis of major risk factors was performed according to the literature (Chen S. et al., 2021). The risk factors for common MDRO infections are currently believed to be age, invasive operation, treatment with three or more antimicrobial agents, and previous multiple or long-term hospitalization. In this study, univariate analyses of 14 common potential risk factors were conducted, and the results are shown in Table 7. Multivariate logistic regression analysis was performed for 6 types of risk factors, and the results suggested that the use of third- or fourth-generation cephalosporins was an independent risk factor for Gram-negative MDRO infections in patients with biliary infection (*p* < 0.05), as shown in Table 8.

**Table 7:** Single factor analysis of characteristics associated with bile Gram-negative MDRO infection in patients with biliary tract infection.

Factors	MDROs (*n* = 1,508)	Non-MDROs (*n* = 832)	*t* value /*Z* value /*χ*^2^ value	*p*-value
Age (Years)	63.78 ± 16.21	60.27 ± 20.75	0.7639^a^	0.529
Hospital stays (Day)	38.51 ± 24.18	18.32 ± 11.83	−5.628^b^	<0.001^*^
Duration of antimicrobial use (Day)	32.74 ± 26.22	13.64 ± 9.25	−3.871^c^	<0.001^*^
Types of antimicrobial agents used (Type)	5.89 ± 2.93	4.40 ± 2.04	−6.267^d^	<0.001^*^
Gender (Male)	1,031	527	0.862^e^	0.599
Use of third or fourth-generation Cephalosporins	1,345	328	0.765^f^	<0.001^*^
Use of Carbapenems	1,440	202	19.77^g^	<0.001^*^
Invasive operation	1,296	197	2.116^h^	<0.001^*^
Ventilation	154	92	1.455^i^	0.122
Operation	1,105	398	0.732^j^	0.650
Associated abdominal infection	56	25	0.701^k^	0.539
Combined diabetes	28	18	0.725^l^	0.676
Complicated with a malignant tumor	67	78	4.670^m^	0.733
Combined with COPD or respiratory failure	122	81	5.222^n^	0.613

Cases with Gram-negative bacilli isolation from 2019 to 2021 were divided into a multidrug-resistant bacteria group and a non-multidrug-resistant bacteria group, and their 14 clinical characteristics were retrospectively analyzed to identify 6 types of potential risk factors. Quantitative variables were expressed as mean and standard deviation (SD). a was the *t* value of the T-test, b–d were the Z value of the Kruskal-Wallis Test, respectively, and e–n were *χ* ^2^ values; COPD, chronic obstructive pulmonary disease. *means statistically significant.

**Table 8:** Multivariate logistic regression analysis of bile Gram-negative MDRO infection in patients with biliary tract infection.

**Risk factors**	Partial regression coefficient	SE	Wald	*p*	OR	95% CI
Hospital stays	−0.025	0.019	0.875	0.634	2.012	0.802–1.670
Duration of antimicrobial use	0.058	0.206	0.927	0.978	0.991	0.877–1.446
Types of antimicrobial agents used	0.534	2.102	2.118	0.331	1.266	0.768–1.885
Third or fourth generation -cephalosporins	1.056	0.202	2.255	0.045^*^	1.908	1.012–2.993
Carbapenems	0.917	0.457	0.501	0.244	1.446	1.047–2.006
Invasive operation	−0.220	0.301	0.128	0.989	1.987	0.529–2.890

Multivariate logistic regression analysis was conducted to assess six types of risk factors, revealing that the use of third- or fourth-generation cephalosporins independently increased the risk of Gram-negative MDRO infections in patients with biliary infection (*p* < 0.05). The asterisk (*) denotes statistical significance.

## 4 Discussion

Bile is a vital bodily fluid that plays a crucial role in digestion and metabolism within the human body. It typically maintains sterility, and samples are frequently collected during routine in-hospital examinations when infected by pathogens (Jang et al., 2023). Unfortunately, its infection is increasing worldwide. When the normal excretion of bile is blocked by tumors, stones, or worm infections, there is increased pressure in the biliary tract, allowing the invasion of intestinal bacteria through the intestine or portal vein. This can promote the development of acute or chronic cholecystitis, as well as abdominal infection (Zhang et al., 2023).

It is well-known that the rates of resistance to antibiotics vary by bacterial type and time (Shen et al., 2023; Yin et al., 2023). The authors of this paper have extensive experience in clinical microbiological detection within hospital settings. For them, gaining a comprehensive understanding of the distribution and drug resistance patterns of common biliary tract pathogens in China is crucial. This knowledge is essential for enhancing detection capabilities and reducing mortality rates among patients with biliary infections.

To address these issues, the intraoperative collection of bile and bacterial culture samples can greatly improve the accuracy of diagnosis and treatment by providing a more comprehensive understanding of the patient’s condition (Asukai et al., 2023; Papazyan et al., 2023). Moreover, although there are many methods to treat corresponding infectious complications, the effect is not satisfactory (Chi et al., 2023). Therefore, it is necessary to actively prevent pathogen infection in patients, and important to understand the characteristics of pathogen infections and their associated risk factors.

At present, there are scientific studies on clinical biliary infection in humans. The 2000 British study and the 2023 India paper reported ambiguity resulting from limited sample size and a lack of detailed pathogen classification (Kar et al., 2023). In contrast, the 2017 report from Japan, as well as the 2006 report of clinical infections in China, identified *Pseudomonas aeruginosa, Enterococcus, Klebsiella pneumoniae,* and *Escherichia coli* as the most prevalent bacteria in biliary tract infection. This aligns with our findings regarding species composition but in an entirely reversed sequence (Chen et al., 2006). Interestingly, the pathogenic dominant strains in our study were similar to those in the Iranian study (Shafagh et al., 2021). There were relatively few controversial studies on pathogen infections in China with the comparison that the dominant strains of acute pancreatic infection were different except for *Escherichia coli* (Chen H. L. et al., 2021). Hence, it is necessary to conduct in-depth research on infectious pathogens in the bile of biliary infection patients.

In this study, the bile samples were incubated and separated by a BacT/Alert3D automated blood culture instrument by international standard operation procedures for bacterial detection (Chen S. et al., 2021); this method can guarantee the growth and accurate identification of specific organisms, even if the samples contained multiple species.

According to the data obtained from bile specimens from 1,556 patients in hospitals. The bile samples in this study had a positive pathogen detection rate of 72.30% during 2019–2021, supporting prior studies conducted in China (Mavri and Smole Mozina, 2013; Wajima et al., 2022). A total of 3,490 pathogenic bacteria were identified, primarily involving Gram-negative bacilli (67.05%), followed by Gram-positive cocci (29.48%) and fungi (3.12%).

During the 3 years, the predominant Gram-negative bacilli included *Escherichia coli*, *Klebsiella pneumoniae*, *Enterobacter cloacae subspecies seweris*, and *Pseudomonas aeruginosa*. The distribution pattern bears a certain degree of resemblance to the microecological arrangement observed in the gastrointestinal tract in SAP (Chen S. et al., 2021). It is noteworthy that *Escherichia coli*, *Klebsiella pneumoniae*, and *Enterobacter cloacae subspecies seweris* exhibited a similar pattern of initial decline followed by an increase; however, their data in 2021 did not surpass that of 2019. In contrast, *Pseudomonas aeruginosa* displayed a distinct pattern of continuous growth. Among the Gram-positive cocci species identified mainly were *Enterococcus faecium* and *Enterococcus faecalis*; however, their trends differed. The former showed a slight decline followed by an increase while the latter demonstrated a decreasing trend. *Candida albicans* was identified as the major fungal species. Regarding obligate anaerobic bacteria, *Clostridium perfringens* remained the primary species but with a declining trend. The presence of fungi and obligate anaerobes detected, however, is relatively limited in quantity. Therefore, there may be some potential deviation, thus it is not necessary to draw excessive inferences.

These findings are consistent with those described in the National Bacterial Resistance Monitoring Network’s monitoring report (Kawecki et al., 2007; Oldereid et al., 2023). *Escherichia coli*, *Klebsiella pneumoniae*, *Enterococcus,* and other intestinal flora were the primary pathogenic Gram-negative bacilli identified by the current study. Biliary tract infection is often caused by opportunistic intestinal pathogens entering retrograde (Babekir et al., 2023), suggesting that they may be related to the placement of drainage tubes, biliary stents, and other interventional treatments (Quinn et al., 2020; Suh et al., 2021; Behroozian et al., 2022).

Most of the bile pathogens in this study were detected in hepatobiliary and pancreatic surgery and tumor surgery departments by the laboratory clinicians. The majority of patients were experiencing inflammation or tumors of the gallbladder, bile duct, liver, or pancreas, and most underwent surgery or interventional treatments, such as ERCP (Mistry and Zeng, 2022). Older age, a higher number of gallstones, hypoproteinemia, recurrent gallstones, relapsed biliary tract surgery, malignant biliary tract infection, and previous antibiotic exposure are all risk factors for biliary tract infection (Gouveia et al., 2017; Kawanishi et al., 2017; Xing et al., 2022). Thus, more attention should be given to these patient populations, including the implementation of intervention measures to prevent biliary tract infection.

Analysis of the Gram-negative bacteria confirmed that *Escherichia coli* and *Klebsiella pneumoniae* Extended-spectrum 𝛽-lactamase (ESBL) detection rates were 37.21 and 17.56%, respectively. While the rate of *Escherichia coli* antibiotic resistance remained stable during 2019–2021, the rate of resistance to ciprofloxacin increased significantly in 2021 (*p* < 0.05). This may be due to the high concentration of this drug in bile. Since ciprofloxacin is a common antibiotic used to treat biliary tract infections, more vigilance is required for the use of this drug. While *Klebsiella pneumoniae* was more susceptible to most antibiotics than *Escherichia coli*, its resistance to tigecycline and carbapenem increased significantly in 2021 (*p* < 0.01).

*Klebsiella pneumoniae* resistance to carbapenem antibiotics was mediated by several different resistance mechanisms, including the production of carbapenemase, increases in effervescent pump activity, and changes in outer membrane protein quantity and function (Yan et al., 2021; Wang et al., 2022; Jing et al., 2023). Tigecycline, one of the newest antibiotic drugs used to treat carbapenem-resistant strains, has been in clinical use in China since 2012 (Fergadaki et al., 2021); however, drug-resistant strains developed quickly and are increasing in number every year. Several mechanisms of resistance, mediated by both chromosomes and plasmids, have been identified (Chen H. L. et al., 2021; Yang J. et al., 2023; Yang Y. et al., 2023). The current study also showed an increase in the resistance of *Klebsiella pneumoniae* to cephalosporins, carbapenems, lactamase inhibitors, cephalases, sminoglycosides, and quinolones (*p* < 0.05). The increase in *Klebsiella pneumoniae* drug resistance indicates a need to intensify the prevention and control of these bacteria in clinical settings.

*Pseudomonas aeruginosa* was one of the primary causes of infection in bile. The rates of resistance to piperacillin, piperacillin/tazobactam, ticacillin/clavulanate, and amikacin decreased significantly over time (*p* < 0.05). After the decline in resistance to quinolones in 2020, however, *Pseudomonas aeruginosa* had varying degrees of resistance in 2021 (*p* < 0.05). This is an important opportunistic pathogen and a causative agent of clinical infections. Patients who are immunocompromised, have complicated underlying diseases, or have received long-term use of antibiotics or interventional therapy are at higher risk of infection and should be monitored more closely to prevent and effectively treat infection (Araya et al., 2023).

Among Gram-positive bacteria, *Enterococcus faecium* was the most likely to develop drug resistance, followed by *Enterococcus faecalis*. The resistance rate of *Enterococcus faecium* to tetracycline increased over time (*p* < 0.05), and strains that were resistant to tigecycline and vancomycin also emerged. Low hemoglobin is significantly associated with the production of vancomycin-resistant enterococci, indicating that drug-resistant bacteria may acquire iron to evolve (Brunson et al., 2023). These findings indicate a need to pay more attention to biliary enterococcal infection in patients with anemia.

While *Enterococcus faecalis* had higher drug sensitivity than *Enterococcus faecium*, the rate of resistance to erythromycin and high-concentration gentamicin decreased over time (*p* < 0.05). Meanwhile, *Enterococcus faecalis* showed no resistance to tigecycline, linezolid, or vancomycin, indicating that these should be the first choice for treatment in severely infected patients. Aminoglycosides can be combined as needed to achieve synergistic effects (Anjum et al., 2022).

*Candida albicans* and *Candida tropicalis* were the top fungal strains isolated from bile, and most were sensitive to common antifungal drugs (Jabeen et al., 2023). These findings indicate that the use of conventional antifungal drugs can have strong therapeutic effects. However, clinicians should make sure to use standardized operating and drug application procedures and strive for aseptic infection.

It is important to note that 12 anaerobic bacteria (0.34%) were also isolated from the bile samples. This finding highlights a need to test bile samples for both aerobic and anaerobic bacteria to avoid missing anaerobic infections. In addition, treatment directed at both aerobic and anaerobic bacteria should be considered for patients with a history of bilio-entero-anastomosis (Ligero-Lopez et al., 2023).

With the extensive use of antimicrobial agents at present, MDROs have become important pathogens of clinical infections. MDROs are resistant to ampicillin, first-generation cephalosporins, and second-generation cephalosporins. Moreover, the sensitivity trends of ceftazidime, piperacillin/tazobactam, cefoperazone/sulbactam, gentamicin, and tobramycin were similar. However, resistance to both ceftriaxone and ciprofloxacin was higher than that to ciprofloxacin. In contrast, resistance to aminoglycosides, cefepime, and carbapenems was higher (Xiao et al., 2023). It was concluded that the drug resistance patterns of MDROs were very different, the patterns of MDROs differ slightly between the northern and southern regions in China, even within the same country (Ruan et al., 2019). This variability is less conducive to clinical anti-infection treatment in patients. Merely, analysis of resistance of MDROs and risk factors for Gram-negative MDRO infection can help identify relevant risk factors and prevent infection (Chen S. et al., 2021). Our results showed that six factors, such as the length of hospital stay, time, antibacterial drug use, antimicrobial type, use of third- or fourth-generation cephalosporins, use of penicillium carbon alkene antimicrobial agents, and invasive operations, were risk factors for Gram-negative MDRO infection in biliary infectious patients. Multivariable logistic regression analysis of these factors indicated that the use of third- or fourth-generation cephalosporins in biliary infectious patients with the presence of biliary Gram-negative MDROs was an independent risk factor for infection and high drug resistance. According to a report by Skorochod et al. (2022), independent risk factors for Israeli patients with *Staphylococcus aureus* hepatobiliary infection included hypertension, bedridden status, and nursing home residence. This suggests that different countries may have their own unique set of independent risk factors for this condition. The rational use of third- or fourth-generation cephalosporins in China was an important measure in preventing MDRO infection in this type of patient.

## 5 Conclusion

In conclusion, this study has determined that Gram-negative bacteria were the predominant pathogens isolated from patients with biliary infections, particularly *Escherichia coli*. The rates and patterns of drug resistance were found to be high and constantly changing. In clinical practice, it is crucial to judiciously use third- or fourth-generation cephalosporins as a key measure to prevent multidrug-resistant organism (MDRO) infections in patients with biliary infections. While our study did not investigate resistance mechanisms, the findings have the potential to inform decision-making in the healthcare sector. Furthermore, this information can be utilized to educate both healthcare professionals and the general public about the importance of appropriate antibiotic use. Additionally, the results of this study could lay a foundation for future research efforts, enabling scientists to explore new detection, therapeutic options, and strategies for biliary infections.

## Data availability statement

The original contributions presented in the study are included in the article/Supplementary material, further inquiries can be directed to the corresponding author.

## Ethics statement

The studies involving humans were approved by Ethical Committee of Nankai Clinical College, Tianjin Medical University. The studies were conducted in accordance with the local legislation and institutional requirements. The ethics committee/institutional review board waived the requirement of written informed consent for participation from the participants or the participants' legal guardians/next of kin because Due to its retrospective design, a waiver of participant informed consent was granted by the Ethical Committee of Nankai Clinical College, Tianjin Medical University.

## Author contributions

SC: Conceptualization, Formal analysis, Funding acquisition, Investigation, Methodology, Project administration, Supervision, Writing – original draft, Writing – review & editing. WL: Data curation, Writing – original draft, Writing – review & editing. XS: Data curation, Funding acquisition, Investigation, Methodology, Writing – original draft, Writing – review & editing. JLu: Data curation, Funding acquisition, Investigation, Methodology, Writing – original draft, Writing – review & editing. JLi: Data curation, Funding acquisition, Investigation, Methodology, Writing – original draft, Writing – review & editing. HO: Data curation, Investigation, Methodology, Writing – original draft, Writing – review & editing. WZ: Data curation, Investigation, Methodology, Writing – original draft, Writing – review & editing. JC: Data curation, Investigation, Methodology, Writing – original draft, Writing – review & editing. ZY: Data curation, Investigation, Methodology, Writing – original draft, Writing – review & editing. HL: Data curation, Investigation, Methodology, Writing – original draft, Writing – review & editing. YZ: Data curation, Investigation, Methodology, Writing – original draft, Writing – review & editing.

## Supplementary material

SUPPLEMENTARY FIGURE S1 The culture images of samples culture and sole bacterial colony isolation in detection. **(A)** The typical aerobic (left) and anaerobic (right) culture bottles with positive indication were shown after aseptically injected by bile samples. **(B)** The Gram-positive bacteria, such as: *Escherichia coli* (Left), *Klebsiella pneumoniae* (middle) and *Pseudomonas aeruginosa* (right) in the detection were displayed. **(C)** The major Gram-positive bacterial as *Enterococcus faecalis* (Left) and *Enterococcus faecium* (right) were compared. **(D)** *Candida albicans* belong with the fungal pathogens were exhibited.

SUPPLEMENTARY FIGURE S2 The number comparison of pathogenic bacteria in patients with bile tract infection in 2019–2021. The graphs displayed case numbers of biliary bacterial infection on the Y-axis and years for bacterial collection on the X-axis. The different graphs represented the following categories of bacteria: **(A)** The Gram-negative bacteria, **(B)** the Gram-positive bacteria, **(C)** the fungal pathogens, and **(D)** the obligate anaerobic bacteria.
