# Supplementary figures and images for: The distribution and antibiotic-resistant characteristics and risk factors of pathogens associated with clinical biliary tract infection in humans
Source: Front Microbiol. 2024 May 9;15:1404366. doi: 10.3389/fmicb.2024.1404366 (PMC11112516; doi:10.3389/fmicb.2024.1404366)

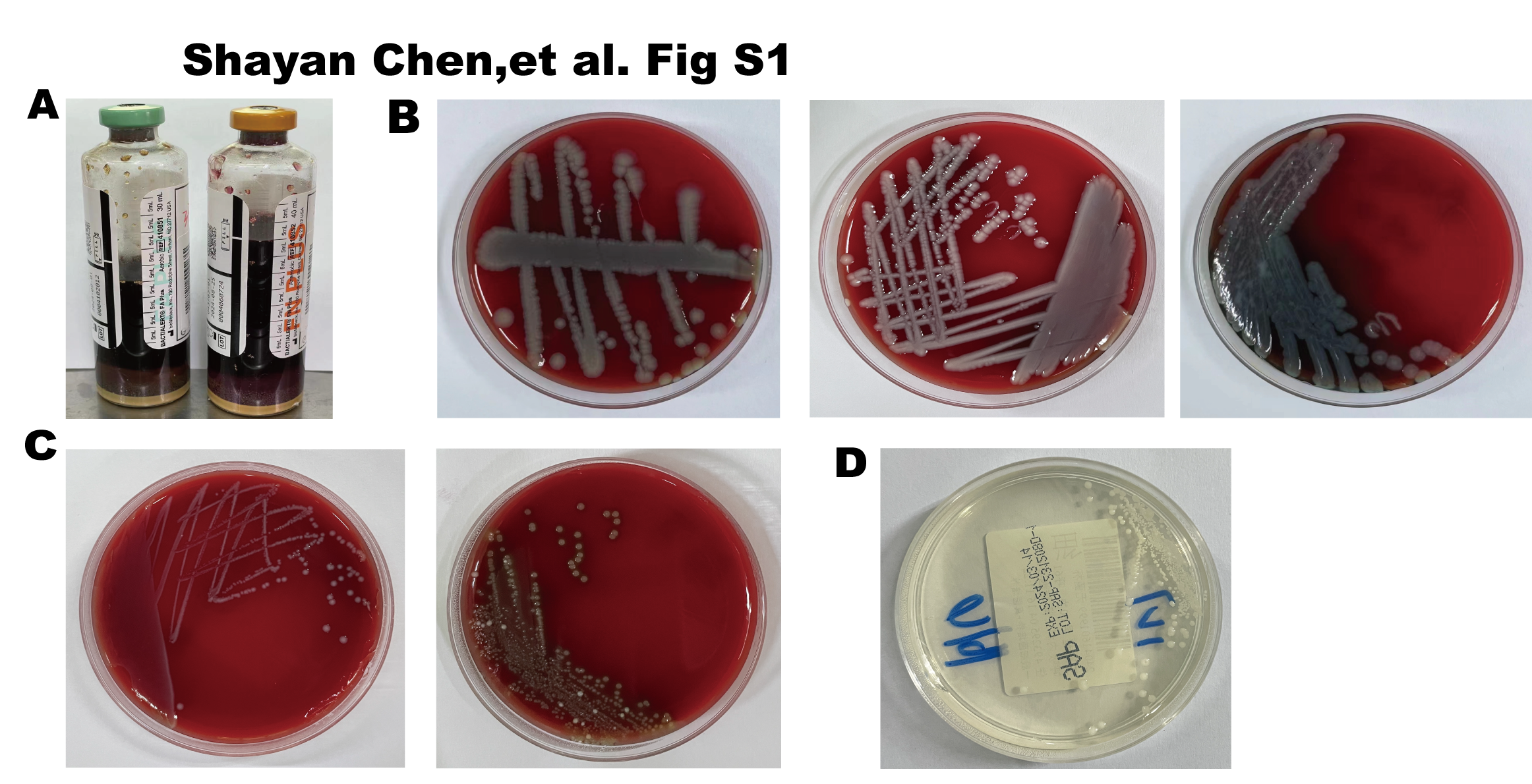

Supplement: SUPPLEMENTARY FIGURE S1 — The culture images of samples culture and sole bacterial colony isolation in detection. (A) The typical aerobic (left) and anaerobic (right) culture bottles with positive indication were shown after aseptically injected by bile samples. (B) The Gram-positive bacteria, such as: Escherichia coli (Left), Klebsiella pneumoniae (middle) and Pseudomonas aeruginosa (right) in the detection were displayed. (C) The major Gram-positive bacterial as Enterococcus faecalis (Left) and Enterococcus faecium (right) were compared. (D) Candida albicans belong with the fungal pathogens were exhibited. [file Image_1.TIF]

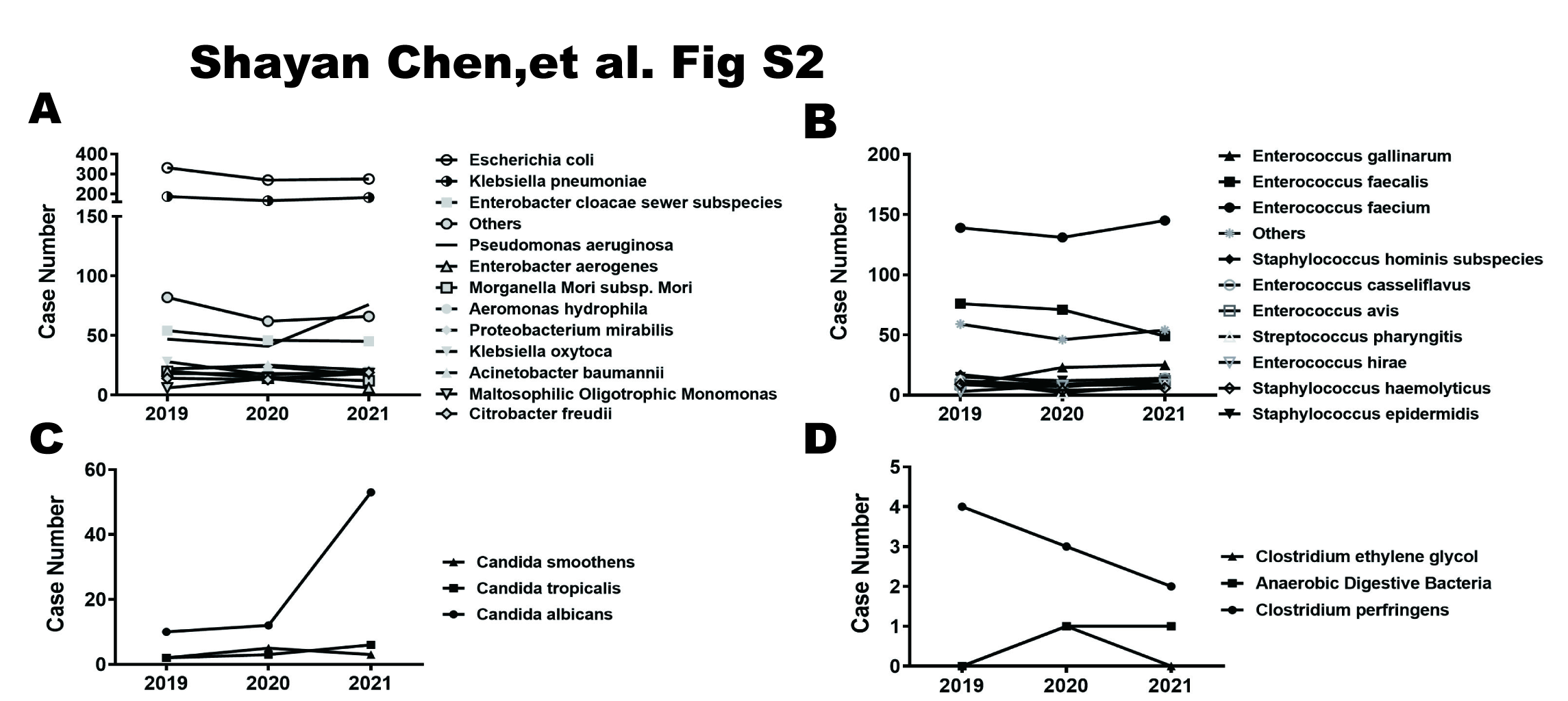

Supplement: SUPPLEMENTARY FIGURE S2 — The number comparison of pathogenic bacteria in patients with bile tract infection in 2019–2021. The graphs displayed case numbers of biliary bacterial infection on the Y-axis and years for bacterial collection on the X-axis. The different graphs represented the following categories of bacteria: (A) The Gram-negative bacteria, (B) the Gram-positive bacteria, (C) the fungal pathogens, and (D) the obligate anaerobic bacteria. [file Image_2.TIF]
